# Detection rates of abnormalities in over 10,000 amniotic fluid samples at a single laboratory

**DOI:** 10.1186/s12884-023-05428-5

**Published:** 2023-02-08

**Authors:** Sha Lu, Nisile Kakongoma, Wen-sheng Hu, Yan-zhen Zhang, Nan-nan Yang, Wen Zhang, Ai-fen Mao, Yi Liang, Zhi-fen Zhang

**Affiliations:** 1 Zhejiang Chinese Medical University, Hangzhou, Zhejiang People’s Republic of China; 2 Prenatal Screening and Prenatal Diagnosis Center, Hangzhou Women’s Hospital (Hangzhou Maternity and Child Health Care Hospital), No. 369 Kunpeng Rd., Hangzhou, Zhejiang 310008 People’s Republic of China; 3 Department of Neurobiology and Acupuncture Research, The Third School of Clinical Medicine, Zhejiang Chinese Medical University, Key Laboratory of Acupuncture and Neurology of Zhejiang Province, 548 Binwen Road, Binjiang District, Hangzhou, 310053 Zhejiang People’s Republic of China

**Keywords:** Prenatal diagnosis, Karyotyping, BoBs assay, SNP array, Amniotic fluid sample

## Abstract

**Background:**

A growing number of cytogenetic techniques have been used for prenatal diagnosis. This study aimed to demonstrate the usefulness of karyotyping, BACs-on-Beads (BoBs) assay and single nucleotide polymorphism (SNP) array in prenatal diagnosis during the second trimester based on our laboratory experience.

**Methods:**

A total of 10,580 pregnant women with a variety of indications for amniocentesis were enrolled in this retrospective study between January 2015 and December 2020, of whom amniotic fluid samples were analysed in 10,320 women. The main technical indicators of participants in the three different technologies were summarized, and cases of chromosome abnormalities were further evaluated.

**Results:**

The overall abnormality detection rate of karyotyping among all the amniotic fluid samples was 15.4%, and trisomy 21 was the most common abnormality (20.9%). The total abnormality detection rate of the BoBs assay was 5.6%, and the diagnosis rate of microdeletion/microduplication syndromes that were not identified by karyotyping was 0.2%. The detection results of the BoBs assay were 100.0% concordant with karyotyping analysis in common aneuploidies. Seventy (87.5%) cases of structural abnormalities were missed by BoBs assay. The total abnormality detection rate of the SNP array was 21.6%. The detection results of common aneuploidies were exactly the same between SNP array and karyotyping. Overall, 60.1% of structural abnormalities were missed by SNP array. The further detection rate of pathogenic significant copy number variations (CNVs) by SNP was 1.4%.

**Conclusions:**

Karyotyping analysis combined with BoBs assay or SNP array for prenatal diagnosis could provide quick and accurate results. Combined use of the technologies, especially with SNP array, improved the diagnostic yield and interpretation of the results, which contributes to genetic counselling. BoBs assay or SNP array could be a useful supplement to karyotyping.

## Background

In recent years, infant mortality and morbidity rates have decreased due to improved standards of living, the implementation of public health measures and the convenience of acquiring knowledge in both developed and developing countries [1, 2]. However, the presence of birth defects worldwide still leads to high infant mortality and morbidity [3, 4], which brings serious financial and psychological burdens to the affected families and society at large [5]. Prenatal diagnosis, such as amniocentesis, is a standard process for detecting and predicting genetic abnormalities and developmental defects in foetuses [6].

Chromosomal abnormalities can appear when there is an anomaly in the structure or number of chromosomes [7]. Conventional karyotyping analysis is the gold standard for prenatal diagnosis of chromosomal abnormalities, although it is considered to be a time-consuming and labour-intensive technique [8]. Superior molecular techniques have been developed to analyse chromosomal abnormalities that are undetectable by karyotyping within 2 weeks, such as microdeletion, microduplication, and copy-number variations (CNVs) [9]. These techniques improve the clinical utility and shorten the detection time, resulting in a faster decision for pregnant women. Such techniques commonly used in our centre include BACs-on-Beads (BoBs) assay, single nucleotide polymorphism (SNP) array and fluorescence in situ hybridization (FISH). The BoBs assay is mainly used for the rapid detection of 5 common chromosome aneuploidies (21, 18, 13, X and Y) and 9 particular microdeletion syndromes [10]. The SNP array method is microarray-based to study the copy number of chromosome and extraordinarily improves the detection of CNVs in the genome [11].

Several studies have compared the diagnostic efficiency of BoBs [12, 13], microarrays [14] and conventional karyotyping [7]. However, those studies were from different laboratories and used various detection platforms and inconsistent reporting criteria. In the current study, we report our experience with karyotyping, BoBs assay and SNP array in over 10,000 cases of amniotic fluid samples analysed in our laboratory. This report also provides useful data on prenatal diagnosis in the prevention and control of birth defects in this region and has important application value for recommending more appropriate cytogenetic technology to pregnant women who request amniocentesis.

## Patients and methods

### Sample collection and preparation

We retrospectively enrolled a cohort of 10,580 participants who required amniocentesis during the second trimester at the Hangzhou Women’s Hospital (Hangzhou Maternity and Child Health Care Hospital) between January 2015 and December 2020. Inclusion criteria in this study were as follows: 1) women with singleton pregnancy; 2) women who chose karyotyping only; 3) women who chose karyotyping and BoBs; 4) women who chose karyotyping and SNP. Pre- and post-test counselling was given by similarly trained clinical genetic counselors in our centre, including the costs, benefits, limitations of the different tests and the laboratory results.

Indications for prenatal diagnosis were as follows: 1) high risk with maternal serum screening (first trimester and/or second trimester): trisomy 21 or trisomy 18; 2) advanced maternal age (age at delivery ≥35 years); 3) high risk of noninvasive prenatal testing (NIPT): trisomy 21, trisomy 18, trisomy 13, sex chromosome abnormality, or other trisomy/microdeletion/microduplication; 4) abnormal ultrasound; 5) previous foetus/child with abnormality; 6) exposure history to chemicals/drug/radiation; 7) abnormal family history/carriers of genetic diseases; 8) chromosomal abnormalities in couples; 9) mixed indications; and 10) other indications: i.e., NIPT failure, preimplantation genetic testing. We analysed different clinical indications in three groups: single indication, two kinds of indications and more than two kinds of indications.

### Karyotyping

Cells from 15 to 20 mL of amniotic fluid were cultured and harvested. According to the standard procedures and the International System for Human Cytogenomic Nomenclature (ISCN), the Giemsa banding technique was performed and analysed. The diagnosis was established within 4 weeks in our laboratory.

### BACs-on-Beads assay

In 2011, Vialard et al. [15] and Gross et al. [16] first evaluated the efficiency of BoBs assay, which is regarded as a rapid cytogenetic technology for prenatal diagnosis [17]. In this assay, amniotic fluid samples do not need to be cultured and the fluid volume of the samples required is less than that required for karyotyping.

Genomic DNA was extracted from 7 to 10 mL of uncultured amniotic fluid using QIAamp DNA Blood Mini Kits (Qiagen, Hilden, Germany). The fluorescence signals of sample DNA were measured using a Luminex 200 instrument system, and the results were analysed by BoBsoft 1.1 software (PerkinElmer Wallac, Turku, Finland). A gain or loss (fluorescence signal higher or lower than that in the reference gene) generates ratios ranging from 1.3 to 1.4 or from 0.6 to 0.8, respectively. The results were completed within 2 weeks.

### Chromosomal single nucleotide polymorphism array

SNP array is a molecular karyotype technology developed in recent years with advantages such as faster turn-around time, less amniotic fluid, unwanted cell culture, higher resolution and superior sensitivity [18]. Many laboratories have applied SNP arrays to prenatal diagnosis as they can gain whole genome information only through DNA and facilitate the detection of microdeletions or microduplications, which overcomes the shortcomings of conventional karyotype analysis [19].

SNP array analysis was performed using the Affymetrix CytoScan 750 K Array (Affymetrix, Santa Clara, CA) according to the manufacturer’s instructions. The results were analysed with Chromosome Analysis Suite software 3.2 (Affymetrix, Santa Clara, CA, USA). All detected CNVs were compared with known CNVs in the scientific literature and with those in the following publicly available databases: Database of genomic variation and Phenotype in Humans using Ensembl Resources (DECIPHER), Database of Genomic Variants (DGV), Online Mendelian Inheritance in Man (OMIM), International Standards for Cytogenomic Array (ISCA) and Global Affymetrix User Pathology Shared Database (CGDB). The results were completed within 2 weeks.

### Statistical analyses

Student’s t test or analysis of variance was used for continuous data, and categorical variables were compared using the Chi-squared analysis. Statistical analysis was performed with SAS System for Windows 9.4 software (SAS Institute Inc., Cary, NC). All tests were two-tailed analyses, and *P* <  0.05 was considered statistically significant.

## Results

### Characteristics of the participants

**Fig. 1:**
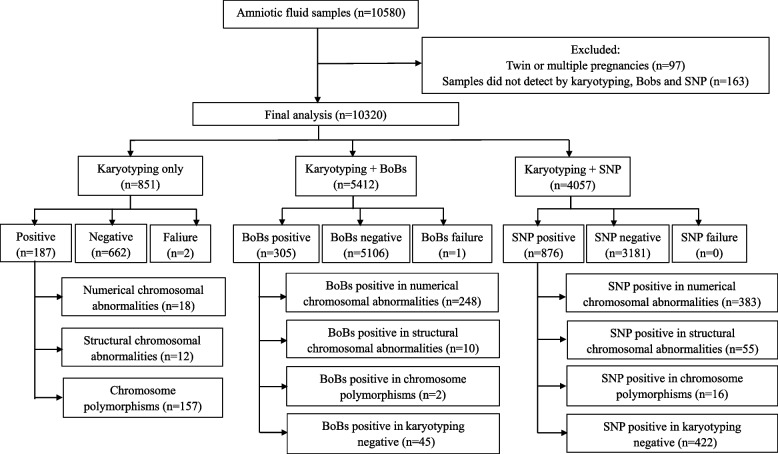
Flow chart of the inclusion of study participants. A total of 10,320 participants were analysed in the study, including 851 cases of choosing karyotyping only, 5412 cases of choosing karyotyping and Bobs, and 4057 cases of choosing karyotyping and SNP

In total, amniotic fluid samples from 10,320 pregnant women were analysed in the study (Fig. 1). As shown in Table 1, 851 pregnant women chose karyotyping only, 5412 chose karyotyping and BoBs, and 4057 chose karyotyping and SNP. Overall, significant differences were found among the three groups in terms of age at delivery, gestational age at invasive testing, husband’s age at delivery, parity and pregnancy outcomes (*P* <  0.01) (Table 1).

**Table 1:** Participants characteristics

	All (*n* = 10,320)	Groups of different choices			*P*
		Karyotyping (*n* = 851)	Karyotyping and BoBs (*n* = 5412)	Karyotyping and SNP (*n* = 4057)	
Age at delivery (years):	32.5 ± 5.6	31.7 ± 5.5	32.8 ± 5.7	32.4 ± 5.5	< 0.001
Gestational age at invasive testing (weeks):	20.1 ± 1.7	20.2 ± 1.6	20.1 ± 1.5	20.2 ± 2.0	0.002
Husband’s age at delivery (years):	34.4 ± 6.2	33.8 ± 5.9	34.7 ± 6.3	34.2 ± 6.2	< 0.001
Parity [n (%)]:					< 0.001
Nulliparous	3688 (35.7)	310 (36.4)	1688 (31.2)	1690 (41.7)	
Outcomes [n (%)]:					< 0.001
Live born	8851 (85.8)	731 (85.9)	4789 (88.5)	3331 (82.1)	
Pregnancy termination or stillbirth	922 (8.9)	31 (3.6)	303 (5.6)	588 (14.5)	
Missed or refused follow-up	547 (5.3)	89 (10.5)	320 (5.9)	138 (3.4)	

The value of *P:* compared among the three groups of different choices

### Chromosomal abnormalities of all specimens in different clinical indications

According to the clinical diagnosis, the proportions for single indication, two kinds of indications and more than two kinds of indications were 80.4% (8300/10320), 18.2% (1883/10320) and 1.3% (137/10320), respectively (Table 2). The most common single indication for amniocentesis was high risk of trisomy 21 in the maternal serum screening test (3366/10320, 32.6%), followed by advanced maternal age (2673/10320, 25.9%) and abnormal ultrasound (927/10320, 9.0%). The total chromosomal anomaly rate, including numerical chromosomal abnormalities, structural chromosomal abnormalities and chromosome polymorphisms was 15.4% (1587/10320). The anomaly rate of each clinical indication is shown in Table 2, with the highest rate being high risk of trisomy 21 in NIPT (79/111, 71.2%), followed by high risk of trisomy 18 in NIPT (19/31, 61.3%) and high risk of sex chromosome abnormality in NIPT (78/176, 44.3%). Except for the indications of high risks in NIPT, the three highest rates of total chromosomal abnormalities were chromosomal abnormalities in couples (10/25, 40.0%), high risk of trisomy 18 in maternal serum screening test (39/194, 20.1%) and exposure history to chemicals/drug/radiation (3/22, 13.6%). Among the numerical chromosomal abnormalities, the top three anomaly rates were consistent with those of the total chromosome abnormalities. Except for the indications of high risks in NIPT, the highest rates of numerical chromosomal abnormalities were high risk of trisomy 18 in the maternal serum screening test (19/194, 9.8%). The highest anomaly rate of structural chromosomal abnormalities was chromosomal abnormalities in couples (9/25, 36.0%). In the chromosomal polymorphisms, the top anomaly rate was exposure history to chemicals/drug/radiation (3/22, 13.6%).

**Table 2:** Chromosomal abnormalities of all specimens in different clinical indications

Clinical indications	Number (proportion %)	Total abnormal (anomaly rate %)	Numerical chromosomal abnormalities (anomaly rate %)	Structural chromosomal abnormalities (anomaly rate %)	Chromosome polymorphisms (anomaly rate %)
**Total**	**10,320**	**1587 (15.4)**	**672 (6.5)**	**230 (2.2)**	**685 (6.6)**
**Total single indication**	**8300 (80.4)**	**1070 (12.9)**	**347 (4.2)**	**152 (1.8)**	**571 (6.9)**
High risk with maternal serum screening					
Trisomy 21	3366 (32.6)	391 (11.6)	56 (1.7)	42 (1.2)	293 (8.7)
Trisomy 18	194 (1.9)	39 (20.1)	19 (9.8)	3 (1.5)	17 (8.8)
Advanced maternal age	2673 (25.9)	265 (9.9)	46 (1.7)	33 (1.2)	186 (7.0)
High risk of NIPT					
Trisomy 21	111 (1.1)	79 (71.2)	76 (68.5)	3 (2.7)	0 (0.0)
Trisomy 18	31 (0.3)	19 (61.3)	18 (58.1)	1 (3.2)	0 (0.0)
Trisomy 13	24 (0.2)	6 (25.0)	3 (12.5)	3 (12.5)	0 (0.0)
Sex chromosome abnormality	176 (1.7)	78 (44.3)	69 (39.2)	5 (2.8)	4 (2.3)
Other trisomy/microdeletion/microduplication	218 (2.1)	34 (15.6)	5 (2.3)	22 (10.1)	7 (3.2)
Abnormal ultrasound	927 (9.0)	106 (11.4)	47 (5.1)	27 (2.9)	32 (3.4)
Previous fetus/child with abnormality	485 (4.7)	36 (7.4)	8 (1.6)	4 (0.8)	24 (5.0)
Exposure history to chemicals/drug/radiation	22 (0.2)	3 (13.6)	0 (0.0)	0 (0.0)	3 (13.6)
Abnormal family history/carriers of genetic diseases	33 (0.3)	3 (9.1)	0 (0.0)	0 (0.0)	3 (9.1)
Chromosomal abnormalities in couples	25 (0.2)	10 (40.0)	0 (0.0)	9 (36.0)	1 (4.0)
Others^a^	15 (0.2)	1 (6.7)	0 (0.0)	0 (0.0)	1 (6.7)
**Two kinds of indications**	**1883 (18.2)**	**476 (25.3)**	**296 (15.7)**	**72 (3.8)**	**108 (5.8)**
**More than two kinds of indications**	**137 (1.3)**	**41 (29.9)**	**29 (21.1)**	**6 (4.4)**	**6 (4.4)**

Proportion % = n / 10,320 × 100%; Anomaly rate % = n / Number× 100%

^a^NIPT failure, preimplantation genetic testing, etc.

### Detailed chromosomal abnormalities detected by karyotyping

Among the 1587 chromosomal abnormalities, 672 cases (42.3%) were numerical chromosomal abnormalities, 230 cases (14.5%) were structural chromosomal abnormalities and 685 cases (43.2%) were chromosome polymorphisms (Table 3). Trisomy 21 was the most common abnormality (331/1587, 20.9%), followed by mosaicism (94/1587, 5.9%), and trisomy 18 (92/1587, 5.8%) in numerical chromosomal abnormalities. The most common aneuploidy in sex chromosomal abnormalities was XXY, followed by XXX, XYY and 45, X.

**Table 3:** Detailed chromosomal abnormalities detected by karyotyping

Abnormality	Number (proportion %)	Fetal gender (anomaly rate %)		Pregnancy outcome (anomaly rate %)		
		Male	Female	Pregnancy termination or stillbirth	Live born	Missed or refused follow-up
**Total**	**1587**	**813 (51.2)**	**774 (48.8)**	**658 (41.5)**	**872 (54.9)**	**57 (3.6)**
**Numerical chromosomal abnormalities**	**672 (42.3)**	**360 (53.6)**	**312 (46.4)**	**579 (86.2)**	**91 (13.5)**	**2 (0.3)**
Trisomy 21	331 (20.9)	194 (58.6)	137 (41.4)	328 (99.1)	3 (0.9)	0 (0.0)
Trisomy 18	92 (5.8)	41 (44.6)	51 (55.4)	92 (100.0)	0 (0.0)	0 (0.0)
Trisomy 13	12 (0.8)	4 (33.3)	8 (66.7)	12 (100.0)	0 (0.0)	0 (0.0)
45, X	18 (1.1)	0 (0.0)	18 (100.0)	17 (94.4)	1 (5.6)	0 (0.0)
47, XXX	38 (2.4)	0 (0.0)	38 (100.0)	13 (34.2)	25 (65.8)	0 (0.0)
47, XXY	61 (3.8)	61 (100.0)	0 (0.0)	47 (77.0)	14 (23.0)	0 (0.0)
47, XYY	22 (1.4)	22 (100.0)	0 (0.0)	8 (36.4)	14 (63.6)	0 (0.0)
Mosaicism of chromosomes	94 (5.9)	41 (43.6)	53 (56.4)	59 (62.8)	33 (35.1)	2 (2.1)
Others^a^	4 (0.3)	1 (25.0)	3 (75.0)	3 (75.0)	1 (25.0)	0 (0.0)
**Structural chromosomal abnormalities**	**230 (14.5)**	**114 (49.6)**	**116 (50.4)**	**66 (28.7)**	**154 (67.0)**	**10 (4.3)**
Translocation	58 (3.7)	29 (50.0)	29 (50.0)	12 (20.7)	45 (77.6)	1 (1.7)
Inversion^b^	99 (6.2)	57 (57.6)	42 (42.4)	3 (3.0)	89 (89.9)	7 (7.1)
Deletion	24 (1.5)	9 (37.5)	15 (62.5)	19 (79.2)	4 (16.7)	1 (4.2)
Duplication	6 (0.4)	3 (50.0)	3 (50.0)	2 (33.3)	4 (66.7)	0 (0.0)
Derivative	29 (1.8)	11 (37.9)	18 (62.1)	20 (69.0)	8 (27.6)	1 (3.4)
Others^c^	14 (0.9)	5 (35.7)	9 (64.3)	10 (71.4)	4 (28.6)	0 (0.0)
**Chromosome polymorphisms**	**685 (43.2)**	**339 (49.5)**	**346 (50.5)**	**13 (1.9)**	**627 (91.5)**	**45 (6.6)**

Proportion % = n / 1587 × 100%; Anomaly rate % = n / Number× 100%

^a^Including 4 cases as follows: 47, XX, +mar; 47, XX, + 9; 48, XXYY; 69, XXX

^b^Including normal variant of inv (9)(p12q13)

^c^Including 14 cases as follows: i/idic (8 cases); ins (1 case); add (2 cases); r (2 cases); trp (1 case)

Between the two foetal gender groups, there was no significant difference in total numerical chromosomal abnormalities, structural chromosomal abnormalities or chromosome polymorphisms (*P* > 0.05) (Table 3).

The rate of pregnancy termination or stillbirth was higher in numerical chromosomal abnormalities than in structural chromosomal abnormalities (86.2% vs. 28.7%, *P* <  0.001), as trisomy 18 and trisomy 13 reached the highest rate (Table 3).

### Abnormalities detected by BoBs assay and karyotyping

In total, 5412 samples were subjected to both karyotyping and BoBs assay, 772 of which were chromosomal abnormalities. The total abnormality detection rate of the BoBs assay was 5.6% (305/5412) (Table 4).

**Table 4:** Detection rate of karyotyping and BoBs assay in 5412 specimens

Abnormality	Detected by karyotyping (proportion %^e^)	BoBs abnormal results (*n* = 305^c^)		Pregnancy outcome (*n* = 817^d^) (proportion %^e^)		
		Detection consistent with the karyotyping (anomaly rate %^f^)	Detected by BoBs only	Pregnancy termination or stillbirth	Live born	Missed or refused follow-up
**Total**	**772**	**258 (33.4)**	**48**	**266 (32.6)**	**519 (63.5)**	**32 (3.9)**
**Numerical chromosomal abnormalities**	**260 (33.7)**	**248 (95.4)**	**0**	**241 (92.7)**	**17 (6.5)**	**2 (0.8)**
Trisomy 21	146 (18.9)	146 (100.0)	0	146 (100.0)	0 (0.0)	0 (0.0)
Trisomy 18	47 (6.1)	47 (100.0)	0	47 (100.0)	0 (0.0)	0 (0.0)
Trisomy 13	5 (0.7)	5 (100.0)	0	5 (100.0)	0 (0.0)	0 (0.0)
45, X	7 (0.9)	7 (100.0)	0	7 (100.0)	0 (0.0)	0 (0.0)
47, XXX	12 (1.6)	12 (100.0)	0	7 (58.3)	5 (41.7)	0 (0.0)
47, XXY	16 (2.1)	16 (100.0)	0	12 (75.0)	4 (25.0)	0 (0.0)
47, XYY	1 (0.1)	1 (100.0)	0	1 (100.0)	0 (0.0)	0 (0.0)
Mosaicism of chromosomes	25 (3.2)	14 (56.0)	0	15 (60.0)	8 (32.0)	2 (8.0)
69, XXX	1 (0.1)	0 (0.0)	0	1 (100.0)	0 (0.0)	0 (0.0)
**Structural chromosomal abnormalities**	**80 (10.4)**	**10 (12.5)**	**1**	**15 (18.8)**	**60 (75.0)**	**5 (6.3)**
Translocation	15 (1.9)	3 (20.0)	0	4 (26.7)	11 (73.3)	0 (0.0)
Inversion	51 (6.6)	0 (0.0)	0	1 (2.0)	46 (90.2)	4 (7.8)
Deletion	3 (0.4)	1 (33.3)	0	3 (100.0)	0 (0.0)	0 (0.0)
Derivative	8 (1.0)	5 (62.5)	0	5 (62.5)	2 (25.0)	1 (12.5)
Others^a^	3 (0.4)	1 (33.3)	1	2 (66.7)	1 (33.3)	0 (0.0)
**Chromosome polymorphisms**	**432 (56.0)**	**0 (0.0)**	**2**^**b**^	**6 (1.4)**	**404 (93.5)**	**22 (5.1)**
**Normal karyotyping**	**0 (0.0)**	**0 (0.0)**	**45**	**4 (8.9)**	**38 (84.4)**	**3 (6.7)**
Sex chromosomal abnormalities	0 (0.0)	0 (0.0)	31	3 (9.7)	26 (83.9)	2 (6.4)
Microdeletion/microduplication	0 (0.0)	0 (0.0)	12	1 (8.3)	10 (83.4)	1 (8.3)
Mosaicism of chromosomes	0 (0.0)	0 (0.0)	2	0 (0.0)	2 (100.0)	0 (0.0)

^a^Including 3 cases as follows: i (1 case); add (2 cases)

^b^Including 2 cases as follows: sex chromosomal abnormalities (1 case); Microdeletion/microduplication (1 case)

^c^If BoBs indicates 2 or more abnormalities in the same amniotic fluid sample, we calculated as 1 case

^d^817 cases = 772 cases of chromosomal abnormalities + 45 cases of BoBs abnormal results in normal karyotyping

^e^Proportion % = n / 772 × 100%

^f^Proportion % = n / 817 × 100%. Anomaly rate % = n / Detected by karyotyping× 100%

The detection results of the BoBs assay were 100.0% concordant with karyotyping analysis in common aneuploidies, including trisomy 21, trisomy 18, trisomy 13 and sex chromosomal abnormalities. In the 25 cases of mosaicism that were detected by karyotyping, only 14 cases (56.0%) were identified by BoBs assay. Two cases of mosaicism, which were verified by BoBs assay only, were further confirmed by interphase FISH and considered false-positive results.

Eighty cases of structural abnormalities were found by karyotyping; however, 70 cases (87.5%) were missed by the BoBs assay. Three translocations that were detected by BoBs assay were unbalanced translocations. We also demonstrated the BoBs results in chromosome polymorphisms. There was no significant difference in the detection rate between the chromosome polymorphisms group (2/432, 0.4%) and the normal karyotyping group (45/4640, 1.0%) (*P* > 0.05).

Only the BoBs assay showed 31 cases of sex chromosomal abnormalities. Twenty-six participants (83.9%) chose to believe the karyotype results and refused further verification. Three participants (9.7%) were worried about possible adverse pregnancy outcomes and directly chose to terminate. The diagnosis rate of microdeletion/microduplication syndromes that were not identified by karyotyping was 0.2% (13/5412).

### Abnormalities detected by SNP array and karyotyping

Overall, 4057 samples were conducted to detect abnormalities by both karyotyping and SNP array (Tables 5 and 6). The total abnormality detection rate of the SNP array was 21.6% (876/4057). Among them, 510 cases (12.6%) of clinically significant variants, 351 cases (8.7%) of variant of unknown significance (VOUS), and 15 (0.4%) cases of LOH were identified. The prevalence of pathogenic CNVs in normal karyotyping was 1.4% (58/4057) (Table 6).

**Table 5:** Detection rate of karyotyping and SNP array in 4057 specimens

Abnormality	Detected by karyotyping (proportion %^d^)	SNP abnormal results (*n* = 454^c^)				Pregnancy outcome (proportion %^e^)		
		Detection consistent with the karyotyping (proportion %^e^)	Detected by SNP only			Pregnancy termination or stillbirth	Live born	Missed or refused follow-up
			Benign	Pathogenic	VOUS			
**Total**	**628**	**438**	**0**	**11**	**75**	**380 (60.5)**	**238 (37.9)**	**10 (1.6)**
**Numerical chromosomal abnormalities**	**394 (62.7)**	**383 (97.2)**	**0**	**1**	**47**	**324 (82.2)**	**70 (17.8)**	**0 (0.0)**
Trisomy 21	178 (28.3)	178 (100.0)	0	1	17	176 (98.9)	2 (1.1)	0 (0.0)
Trisomy 18	42 (6.7)	42 (100.0)	0	0	2	42 (100.0)	0 (0.0)	0 (0.0)
Trisomy 13	7 (1.1)	7 (100.0)	0	0	0	7 (100.0)	0 (0.0)	0 (0.0)
45, X	10 (1.6)	10 (100.0)	0	0	0	9 (90.0)	1 (10.0)	0 (0.0)
47, XXX	24 (3.8)	24 (100.0)	0	0	1	4 (16.7)	20 (83.3)	0 (0.0)
47, XXY	44 (7.0)	44 (100.0)	0	0	2	34 (77.3)	10 (22.7)	0 (0.0)
47, XYY	21 (3.3)	21 (100.0)	0	0	1	7 (33.3)	14 (66.7)	0 (0.0)
Mosaicism of chromosomes	66 (10.5)	55 (83.3)	0	0	24	43 (65.2)	23 (34.8)	0 (0.0)
Others^a^	2 (0.3)	2 (100.0)	0	0	0	2 (100.0)	0 (0.0)	0 (0.0)
**Structural chromosomal abnormalities**	**138 (22.0)**	**55 (39.9)**	**0**	**9**	**13**	**50 (36.2)**	**86 (62.3)**	**2 (1.5)**
Translocation	41 (6.5)	4 (9.8)	0	0	6	7 (17.1)	33 (80.5)	1 (2.4)
Inversion	39 (6.2)	0 (0.0)	0	0	1	2 (5.1)	37 (94.9)	0 (0.0)
Deletion	21 (3.3)	20 (95.2)	0	0	0	16 (76.2)	4 (19.1)	1 (4.8)
Duplication	6 (1.0)	6 (100.0)	0	0	0	2 (33.3)	4 (66.7)	0 (0.0)
Derivative	21 (3.3)	15 (71.4)	0	9	6	15 (71.4)	6 (28.6)	0 (0.0)
Others^b^	10 (1.6)	10 (100.0)	0	0	0	8 (80.0)	2 (20.0)	0 (0.0)
**Chromosome polymorphisms**	**96 (15.3)**	**0 (0.0)**	**0**	**1**	**15**	**6 (6.3)**	**82 (85.4)**	**8 (8.3)**

^a^Including 2 cases as follows: 47, XX, + 9; 48, XXYY

^b^Including 10 cases as follows: i/idic (6 cases); ins (1 case); r (2 cases); trp (1 case)

^c^If SNP indicates 2 or more abnormalities in the same amniotic fluid sample, we calculated as 1 case

^d^Proportion % = n / 628 × 100%

^e^Proportion % = n / Detected by karyotyping× 100%

**Table 6:** Detailed abnormalities detected by SNP array in normal karyotyping

Abnormality	Number (proportion %^a^)	Fetal gender (proportion %^b^)		Pregnancy outcome (proportion %^b^)			Parental testing				
		Male	Female	Pregnancy termination or stillbirth	Live born	Missed or refused follow-up	Testing number (proportion %^b^)	Maternal (proportion %^c^)	Paternal (proportion %^c^)	De novo (proportion %^c^)	Parental (proportion %^c^)
**Total**	**422**	**236 (55.9)**	**186 (44.1)**	**68 (16.1)**	**337 (79.9)**	**17 (4.0)**	**266 (63.0)**	**136 (51.1)**	**95 (35.7)**	**34 (12.8)**	**1 (0.4)**
**Mosaicism**	**13 (3.1)**	**5 (38.5)**	**8 (61.5)**	**6 (46.2)**	**7 (53.8)**	**0 (0.0)**	**0 (0.0)**	**0 (0.0)**	**0 (0.0)**	**0 (0.0)**	**0 (0.0)**
**Pathogenic**	**58 (13.7)**	**40 (69.0)**	**18 (31.0)**	**19 (32.8)**	**37 (63.8)**	**2 (3.4)**	**29 (40.9)**	**12 (41.4)**	**3 (10.3)**	**14 (48.3)**	**0 (0.00)**
Microduplication	9 (2.1)	7 (77.8)	2 (22.2)	4 (44.4)	4 (44.4)	1 (11.2)	5 (55.6)	2 (40.0)	1 (20.0)	2 (40.0)	0 (0.0)
Microdeletion	46 (10.9)	30 (65.2)	16 (34.8)	15 (32.6)	30 (65.2)	1 (2.2)	24 (52.2)	10 (41.7)	2 (8.3)	12 (50.0)	0 (0.0)
Microdeletion and microduplication	3 (0.7)	3 (100.0)	0 (0.0)	0 (0.0)	3 (100.0)	0 (0.0)	0 (0.0)	0 (0.0)	0 (0.0)	0 (0.0)	0 (0.0)
**VOUS**	**336 (79.6)**	**186 (55.4)**	**150 (44.6)**	**40 (11.9)**	**281 (83.6)**	**15 (4.5)**	**235 (69.9)**	**124 (52.8)**	**92 (39.1)**	**18 (7.7)**	**1 (0.4)**
Microduplication	195 (46.2)	97 (49.7)	98 (50.3)	18 (9.2)	170 (87.2)	7 (3.6)	133 (68.2)	75 (56.4)	54 (40.6)	4 (3.0)	0 (0.0)
Microdeletion	127 (30.1)	82 (64.6)	45 (35.4)	15 (11.8)	104 (81.9)	8 (6.3)	95 (74.8)	48 (50.5)	36 (37.9)	11 (11.6)	0 (0.0)
Microdeletion and microduplication	14 (3.3)	7 (50.0)	7 (50.0)	7 (50.0)	7 (50.0)	0 (0.0)	7 (50.0)	1 (14.3)	2 (28.6)	3 (42.8)	1 (14.3)
**LOH**	**15 (3.6)**	**5 (33.3)**	**10 (66.7)**	**3 (20.0)**	**12 (80.0)**	**0 (0.0)**	**2 (13.3)**	**0 (0.0)**	**0 (0.0)**	**2 (100.0)**	**0 (0.0)**

^a^Proportion % = n / 422 × 100%

^b^Proportion % = n / Number × 100%

^c^Proportion % = n / Testing number × 100%

The detection results of common aneuploidies were exactly the same between SNP array and karyotyping, including autosomal trisomy (21, 18, 13, 9), sex chromosomal monosomy and sex chromosomal trisomy. In the 66 cases of mosaicism that were detected by karyotyping, 55 (83.3%) were identified by SNP array (Table 5).

In normal karyotyping cases, 422 cases were detected by SNP array only. In 13 cases of mosaicism, which were verified by SNP only, 3 cases were further confirmed by interphase FISH and 1 case was considered a false-positive result. Five participants refused further verification and decided to terminate pregnancy. The remaining 5 cases were 47, XXX/46, XX, and chose to continue pregnancy (Table 6).

A total of 266 cases (266/422, 63.0%) chose further parental testing, including 136 cases (136/266, 51.1%) of maternal inheritance, 95 cases (95/266, 35.7%) of paternal inheritance and 34 cases (34/266, 12.8%) of de novo foetal mutations. The rate of maternal inheritance was higher than that of paternal inheritance. The rate of pregnancy termination or stillbirth was higher in the pathogenic group than in the variant of unknown significance (VOUS) group (*P* <  0.01), while rate of further parental testing was lower (*P* <  0.05) (Table 6).

## Discussion

The current study demonstrated the results of prenatal diagnosis in our laboratory, which had the standardised doctor training, standardised definition of prenatal diagnosis indication, standardised test conditions and consistent reporting criteria. Our results could be used as important data on prenatal diagnosis in the prevention and control of birth defects for the local government and doctors, and may have fundamental application value for recommending more appropriate cytogenetic technology to pregnant women who request amniocentesis.

It was reported that in China the prevalence of birth defects is about 5.6%, with chromosomal abnormalities accounting for an important proportion [20]. Prior studies have implied that trisomy 21 is the most common chromosomal abnormality of birth defects, followed by sex chromosomal abnormalities and trisomy 18 [21–23]. In our study, trisomy 21 was also the most common abnormality, followed by mosaicism, and trisomy 18 in numerical chromosomal abnormalities in the second trimester. The most common aneuploidy in sex chromosomal abnormalities was XXY, followed by XXX, XYY and 45, X.

For pregnant women with prenatal diagnostic indications, the preferred recommendation of our centre is amniocentesis. However, with the development of NIPT, some pregnant women with a high risk of birth defects refused our first recommendation and chose NIPT; this is because of the fear of amniocentesis as well as the high accuracy of NIPT in publicity materials (> 95.0% accurate) [24]. Pregnant women often ask, “Doctor, is amniocentesis necessary for me?” Therefore, we showed the probability of chromosomal abnormalities in different prenatal diagnostic indications to provide better guidance. As expected, the anomaly rate of the more than two kinds of indications group was higher than that of the two kinds of indications group and the single indication group. In general, the anomaly rates of single indications in numerical chromosomal abnormalities were all higher than 1.0%, except for the indication of the exposure history to chemicals/drug/radiation, abnormal family history/carriers of genetic diseases, chromosomal abnormalities in couples and others. Based on a recent meta-analysis, the pregnancy loss rate after amniocentesis procedures has been estimated at 0.3-0.6% [25–27], and the risk in our centre is 0.1%. Therefore, amniocentesis has more advantages than disadvantages and can be necessary for pregnant women with prenatal diagnostic indications.

Karyotyping has the advantage of detecting chromosomal numerical and structural abnormalities, including translocation, inversion, duplication and deletion (greater than about 5 Mb) [28]. It is highly sensitive and specific, and exceptionally reliable for identifying foetal chromosomal abnormalities [29]. According to the karyotyping results, couples can always make a quick decision about whether or not to terminate the pregnancy. Women with trisomy 18 and trisomy 13 reached the highest rates of pregnancy termination or stillbirth chromosomal abnormalities in our centre, followed by trisomy 21. However, the main disadvantage of this technology for couples is the requirement of a longer time to obtain results, which can lead to increased anxiety levels during pregnancy. Meanwhile, karyotyping demands high-level technicians to process the samples and interpret the chromosome results and has the possibility of cultivating failure, which means that no results can be provided.

Several studies demonstrated that there were no false-positive or false-negative results detected by BoBs assay compared with karyotyping analysis for common aneuploidies [12, 13, 30]. A normal BoBs result can greatly reduce psychological stress in couples. To evaluate the efficiency of the BoBs assay, we also compared the results with traditional karyotyping in the same enrolled pregnant women. The results of our study of 5412 samples further ensured the improvement of the diagnostic ability of common aneuploidy (trisomy 21, trisomy 18, trisomy 13) by using BoBs assay compared to karyotyping. However, for sex chromosomal abnormalities, there were 67 cases detected by BoBs assay and only 36 (53.7%) were completely concordant with karyotyping analysis. Additionally, BoBs assay detected 13 cases of microdeletion or microduplication, which were not identified by karyotyping, allowing an additional diagnostic result of 0.2% for microdeletion or microduplication syndromes. Similar to our results, Li et al. reported that the additional diagnostic rate of those syndromes was 0.2% [13]. Tao et al. identified that the chromosomal microduplications/microdeletions rate of BoBs was 1.9% [12], and another study showed that the rate was 1.6% [30]. However, BoBs missed 11 (44.0%) mosaic cases, which might have been because of technical limitations on the ability to identify mosaics in different targeted regions [31]. In addition, two cases of mosaicism verified by BoBs assay only were further confirmed by interphase FISH and considered false-positive results, which might be connected to placental mosaicism, maternal cell contamination or other uncertain laboratory factors [31]. In structural chromosomal abnormalities, BoBs assay failed to detect inversions and balanced translocations. In general, BoBs assay is more rapid, easier, labour-saving, and has additional detection for microdeletion/microduplication syndromes [17]. Couples can choose BoBs assay to obtain more rapid results for trisomy 21, trisomy 18 and trisomy 13 to reduce psychological pressure.

Chromosomal microarray analysis is the first recommendation for pregnant women with ultrasound abnormalities [32]; however, pathogenic CNVs can also be detected in foetuses with normal ultrasound. Wapner et al. enrolled 4406 women and demonstrated an increased diagnostic rate in microarray over karyotyping of 6.0% among cases with ultrasound abnormalities and 1.7% among pregnant women with advanced maternal age or positive screening results [33]. A 2020 study indicated that the frequency of pathogenic or likely pathogenic CNVs is 1.2% in all indications by prenatal SNP array [34]. A recent review summarized a total of 29,612 cases using array techniques in foetuses with abnormal structures and the rate of pathogenic or likely pathogenic CNVs was 0.4–2.5% [35]. In our study, we found a 1.4% increased diagnostic yield, which might have been missed if only conventional karyotyping had been performed. The incidence shown in our study was lower than that in some studies, which could possibly be related to the lower incidence of abnormal ultrasound findings before 24 gestational weeks. Meanwhile, some pregnant women with abnormal ultrasound chose to terminate the pregnancy first and use the samples from the foetus to check chromosome abnormalities, which were not included in this study.

In our study, the SNP array results were consistent with all the numerical chromosomal abnormalities of karyotyping analysis except 11 (2.8%, 11/394) cases of mosaicism. Furthermore, the SNP array detected 409 cases of chromosomal microdeletion, microduplication and LOH, with an additional diagnostic rate of 10.1% (409/4057). In the 66 cases of mosaicism that were detected by karyotyping, 55 (83.3%) were identified by SNP array, showing a superior detection ability compared to BoBs. In 13 cases of mosaicism, which were verified by SNP only, 3 cases were further confirmed by interphase FISH, and 1 case was considered a false-positive result. Five participants refused further verification and decided to terminate the pregnancy. The remaining 5 cases were 47, XXX/46, XX, and chose to continue pregnancy. Although it was originally controversial whether SNP array analysis could identify mosaicism, array techniques appear to be more sensitive than conventional karyotyping in detecting low-level mosaicism [36, 37]. Schaeffer et al. also showed a higher detection rate of mosaicism in microarray than in karyotyping [38]. It has been suggested that the difference in cell proliferation with various karyotypes might lead to inconsistencies between cultured samples (karyotyping) and uncultured samples (SNP array) under in vitro cell culture. Cell culture, reported to increase with the age of the culture, tended to promote the growth of euploid cells rather than aneuploid cells [39].

The results of the SNP array did influence the pregnancy outcomes, whether pathogenic CNVs or VOUS. In the cases of foetuses with pathogenic CNVs, 19 (32.8%) couples chose to terminate the pregnancy, although several cases were verified to be inherited from the parents. The diagnosis of VOUS brings challenges in genetic counselling and causes couples’ psychological anxiety. The rate of pregnancy termination in foetuses with VOUS was 11.9%, and most of the reasons were due to abnormal ultrasound. However, 17 (5.1%) cases in the VOUS group feared the uncertainties and stubbornly decided to terminate the pregnancy. Thus, for VOUS foetuses, further long-term follow-up studies, experience accumulation and sharing are necessary [40].

In general, SNP array and karyotyping each have their own advantages in chromosome analysis. SNP arrays are more sensitive for detecting CNVs, and karyotyping is useful for detecting chromosomal balance recombination. The two technologies should not be replaced because their combination can provide more information and optimize genetic counselling.

Although chromosome polymorphism is an abnormal result in karyotyping, many normal adults also have chromosome polymorphisms. We are interested in whether chromosome polymorphism is abnormal in BoBs assay or SNP array. As the results showed, there were no significant differences in the detection rate between the chromosome polymorphisms group and the normal karyotyping group in either BoBs assay or SNP array. The latest version of ISCN 2020 recommended that chromosome polymorphisms should not be included in ISCN nomenclature descriptions in order to avoid misinterpretation [41]. Our study also supports this conclusion.

This study has some limitations. First, some results could not be better evaluated due to the participants’ refusal of further validation or providing information about pregnancy outcomes. Second, the study lacked information on postnatal long-term follow-up of foetuses carrying CNVs. We will perform a long-term follow-up of the offspring and evaluate the data. Third, there was a limited number of some clinical indications. Therefore, further studies will continue to accumulate data and more clinical indications can be conducted to confirm our findings.

## Conclusions

In conclusion, karyotyping analysis combined with BoBs assay or SNP array for prenatal diagnosis can provide quick and accurate results. Combined use of the technologies, especially with SNP array, undoubtedly improved the diagnostic yield and interpretation of the results, which can contribute to genetic counselling. If couples can afford the SNP array, we suggest that it could be recommended to all women undergoing amniocentesis regardless of the indication of the prenatal diagnosis.

## Data Availability

The datasets used for this study are publically available in the OSF support website (https://osf.io/u4ztd/).

## Abbreviations

BoBs: BACs-on-Beads
CNVs: Copy number variations
FISH: Fluorescence in situ hybridization
ISCN: International System for Human Cytogenomic Nomenclature
NIPT: Noninvasive prenatal testing
SNP: Single nucleotide polymorphism
VOUS: Variant of unknown significance
